# Stiff-person Syndrome and GAD Antibody-spectrum Disorders: GABAergic Neuronal Excitability, Immunopathogenesis and Update on Antibody Therapies

**DOI:** 10.1007/s13311-022-01188-w

**Published:** 2022-01-27

**Authors:** Marinos C. Dalakas

**Affiliations:** 1 Department of Neurology, Thomas Jefferson University, Philadelphia, PA USA; 2 Neuroimmunology Unit National and Kapodistrian University of Athens Medical School, Athens, Greece

## Abstract

Although antibodies against Glutamic Acid Decarboxylase (GAD) were originally associated with Stiff Person Syndrome (SPS), they now denote the *“GAD antibody-spectrum disorders (GAD-SD)”* that include Cerebellar Ataxia, Autoimmune Epilepsy, Limbic Encephalitis, PERM and eye movement disorder. In spite of the unique clinical phenotype that each of these disorders has, there is significant overlapping symptomatology characterized by autoimmune neuronal excitability. In addition to GAD, three other autoantibodies, against glycine receptors, amphiphysin and gephyrin, are less frequently or rarely associated with SPS-SD. Very high serum anti-GAD antibody titers are a key diagnostic feature for all GAD-SD, commonly associated with the presence of GAD antibodies in the CSF, a reduced CSF GABA level and increased anti-GAD-specific IgG intrathecal synthesis denoting stimulation of B-cell clones in the CNS. Because anti-GAD antibodies from the various hyperexcitability syndromes recognize the same dominant GAD epitope, the clinical heterogeneity among GAD-SD patients remains unexplained. The paper highlights the biologic basis of autoimmune hyperexcitability connected with the phenomenon of reciprocal inhibition as the fundamental mechanism of the patients’ muscle stiffness and spasms; addresses the importance of high-GAD antibody titers in diagnosis, pinpointing the diagnostic challenges in patients with low-GAD titers or their distinction from functional disorders; and discusses whether high GAD-antibodies are disease markers or pathogenic in the context of their association with reduced GABA level in the brain and CSF. Finally, it focuses on therapies providing details on symptomatic GABA-enhancing drugs and the currently available immunotherapies in a step-by-step approach. The prospects of future immunotherapeutic options with antibody therapies are also summarized.

**Supplementary Information:**

The online version contains supplementary material available at 10.1007/s13311-022-01188-w.

## Introduction

Autoantibodies against Glutamic Acid Decarboxylase (GAD), the rate-limiting enzyme for the synthesis of the inhibitory gamma-aminobutyric acid (GABA), originally seen in patients with Stiff Person Syndrome (SPS), epilepsy and Type-1 Diabetes Mellitus (DM-1) [1, 2], are now connected with several neurological autoimmunities characterized by neuronal excitability comprising the *“GAD antibody-spectrum disorders (GAD-SD)”* [3–8]. This disease spectrum includes in addition to SPS, Autoimmune Epilepsy, Cerebellar Ataxia, Limbic Encephalitis, Myoclonus and Nystagmus [3–9]. As GAD is widely expressed not only within the central nervous system but also the pancreatic β-cells, anti-GAD antibodies have highlighted from the outset an immunological connection between autoimmune neuronal excitability disorders and DM-1 [2]. Approximately 30% of GAD-SD patients also have DM1 while low-titer anti-GAD antibodies are also found in about 80% of patients with DM1 [3, 7, 8]. In contrast to anti-GAD-SD, however, where high-titer antibodies are distinctly against linear epitopes, in DM1 the low-anti-GAD antibodies are directed against conformational epitopes [3–9].

The article describes the clinical spectrum of GAD-antibody-associated disorders as have now evolved, stressing their overlapping symptomatology while highlighting various puzzling clinical connections, diagnostic challenges or pathogenetic mechanisms. It discusses how impaired GABAergic neurotransmission results in diverse clinical phenomena; stresses the importance of reciprocal inhibition in muscle stiffness; outlines the importance of GAD antibody titers in defining the GAD-SD; and summarizes the best therapeutic options in treating autoimmune neuronal excitability. At the practical level, the paper aims to enhance awareness of these syndromes useful to the practicing neurologists in facilitating diagnosis and provides a step-by-step therapeutic scheme from disease initiation to further progression. Considering that SPS is a potentially treatable disorder but remains still misdiagnosed or overdiagnosed based on the patients referred to our clinic, early recognition is critical for prompt therapy initiation.

## Evolution of GAD-SD: a 65-Year Evolution after the Initial Description

The GAD-SD began with *Stiff-Man Syndrome (SMS)*, described by Moersch and Woltman in 1956, characterized by muscle rigidity, hyperreflexia and spasms, mainly in the truncal and proximal leg muscles with excellent response to diazepam [1]. The second hallmark observation was the description of autoantibodies against GAD by Solimena et al. [2] who detected anti-GAD antibodies in both serum and cerebrospinal fluid, pointed out an immunological connection between SMS and DM1 and concluded that the clinical manifestations of SMS are related to disruption of GABAergic pathways [2], a notion still valid today. Subsequently, autoantibodies against synaptic proteins were described, first against amphiphysin in *“three women with the stiff-man syndrome and breast cancer”* [10, 11] and then against gephyrin in one SMS patient [12]. On the clinico-neurophysiological side, understanding of SMS progressed with the description of abnormal excitotoxicity and paroxysmal autonomic dysfunction [13–15], recognition of clinical heterogeneity [4, 7–9], and subdividing SMS into stiff trunk (man) syndrome, stiff limb syndrome and progressive encephalomyelitis with rigidity [16].

It was in 1999–2000 when in the largest at that time series of examined patients, the clinical spectrum and diagnostic criteria valid today were described [3], while the term *Stiff-Person Syndrome (SPS*) was introduced based on the observations that more patients were women, hence the more appropriate term “person”-rather than “man” [3, 17–19], especially since in previous publications it was referred even in the title as *“three women with stiff-man syndrome”* [10]. Over the ensuing years, the pathomechanism of SPS was further characterized with novel electrodiagnostic neuronal excitability studies [19–21]; GABA measurements in the CSF [18] and brain with MRS spectroscopy [22]; immunological studies including GAD epitopes and search for other antibodies affecting GABAergic neurotransmission [23–26]; performance of two controlled clinical trials [27, 28]; and defining the natural history of the disease based on the largest series of SPS patients examined by the same clinicians longitudinally over a 20 year period [29].

Over the years, it became also apparent by many investigators in the field, as discussed later, that since GAD is widely expressed within the central nervous system catalyzing the conversion of the excitatory neurotransmitter l-glutamate to the inhibitory gamma-aminobutyric acid (GABA), anti-GAD antibodies are also associated with other autoimmune neurological diseases manifested by neuronal excitability. These GAD-associated syndromes, all characterized by abnormal synaptic neurotransmission, comprise the “GAD antibody-spectrum disorders (GAD-SD)” or “SPS-SD” since SPS remains the hallmark disease among all of them, although one or more of these disorders coexists in approximately 70% of patients with GAD65 neurological autoimmunity [3, 6–9, 30–38].

## GAD-SD: Clinical Spectrum, Importance of Anti-GAD Antibody Titers, CSF Characteristics and Autoimmune Neuronal Excitability

In all GAD-SD, there is impaired GABAergic neurotransmission resulting in neuronal excitability, presumably by the GAD-targeting antibodies. In spite of their overlapping symptomatology, however, each disease within the spectrum maintains a distinct phenotype. In a recent large retrospective record review of 212 GAD65 neurological autoimmunity samples examined at the Mayo clinic laboratory from 2003–2018, 50% had SP-SD, 43% cerebellar ataxia, 29% autoimmune epilepsy and 16% limbic encephalitis [38]. These frequencies are also consistent with our experience. The clinical characteristics of each disease subtype and their GAD-associated pathogenicity that collectively define the GAD-SD are as follows:

### Stiff Person Syndrome (SPS)

**Fig. 1:**
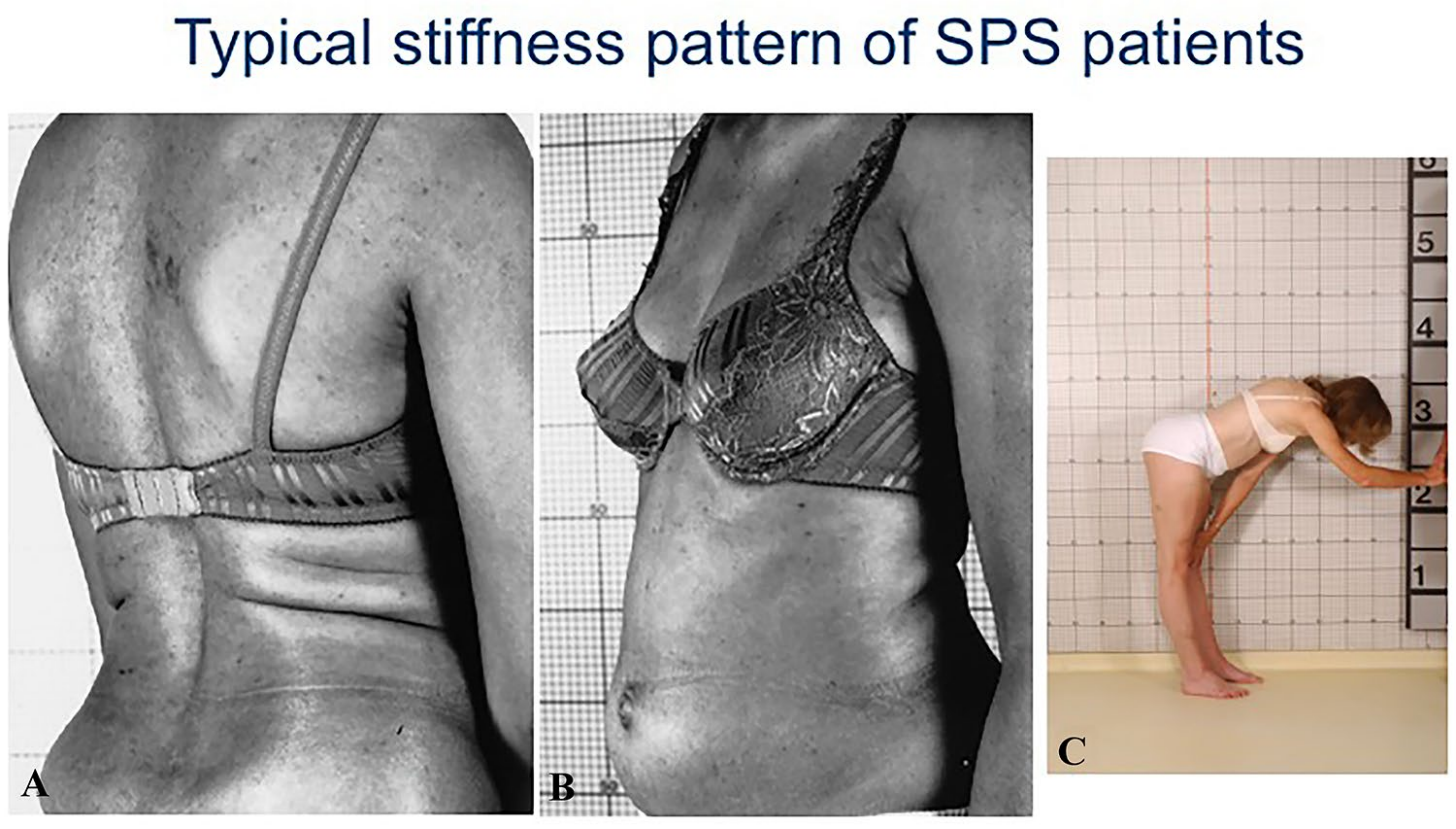
Typical stiffness pattern in SPS patients **A-C:** Concurrent stiffness of agonists (thoracolumbar A) and antagonists (abdominal B) muscles due to lack of reciprocal inhibition in a patient with typical Stiff-Person Syndrome Another patient (C) shows significant stiffness with inability to bend forwards (modified from Dalakas et al. [3])

Although said to affect approximately 1 in a million people [39], the precise frequency and estimated SPS prevalence are unclear, especially when viewed within the GAD spectrum disorders. As judged by the large number of patients referred to us and personally examined and followed the last 30 years, we believe SPS is more common than previously thought but still under-recognized. It is twice as common in women than men above the age of 20 years [3, 8, 17–19, 33–36]. Patients typically present with muscle spasms and stiffness, concurrently in the thoracolumbar paraspinal and abdominal muscles, resulting in difficulty turning and bending (Fig. 1). When stiffness is severe, the patients’ walking resembles a “statue” or exhibits a “freezing-like” appearance; some patients mention that they walk like a “tin-man” with hyperlordotic posture [3, 9, 17–19]. Spasms are frequent in the trunk or any extremity and can be painful. Spasms can be also seen in the face and if limited exclusively to the facial muscles, as we have seen in rare patients with very high GAD-antibody titers, we have referred it as *“stiff-face syndrome.”* Muscle spasms and stiffness can be precipitated by unexpected stimuli, including sounds, like a phone ring or a siren, sudden touches or conditions triggering anxiety and emotional upset which, when severe, are misdiagnosed as a primary anxiety disorder. The episodic nature of spasms, often emphasized by the patients, is important to acknowledge because at times it may not be obvious when a patient is first examined but may become apparent even 30 min later as the patient becomes more anxious even while waiting in the examining room. Task-specific phobias, especially fear of walking, crossing a street or a green light and fear of falling, are quite common [3, 9, 17–19, 26, 27, 33–35]. In some cases, these or similar events can cause severe and continuous painful spasms, along with stiffness in the thoracic muscles with breathing difficulties, tachycardia and hyperhidrosis, a condition we have labeled “status spasticus,” requiring emergency admission for intravenous diazepam [9, 33–35]. Electrophysiological studies reveal continuous activity of motor unit firing at rest, confirming that stiffness is caused by co-contractions of agonists and antagonists muscles [3, 9, 19–21].

#### Diagnosis

The diagnostic criteria for SPS as revised in 2001 [3, 17–19] include the constellation of: a) stiffness of the axial muscles, especially abdominal and thoracolumbar paraspinals, leading to hyperlordosis (Fig. 1); b) superimposed painful spasms triggered by anxiety, tactile or auditory stimuli; c) electromyographic evidence of continuous motor unit activity of agonist and antagonist muscles; d) high-titer GAD antibodies with the cut-off positivity titers > 10.000 IU/ml by ELISA. In patients with suspected SPS-SD but with anti- GAD antibody-titer lower than 10,000, a spinal tap is necessary to assess GAD positivity in the CSF [9, 37]; and e) absence of other neurological findings that may suggest an alternative diagnosis. Exclusion of functional disorders is an important consideration in patients with low or negative anti-GAD antibody-titers posing diagnostic challenge in approximately 20% of such patients frequently seen in specialty clinics. In seronegative patients, adherence to strict clinical SPS criteria, neurophysiologic testing and neuropsychiatric examination are essential. An empirical trial with diazepam is often used for relieve of spasms, but it does not ensure diagnostic accuracy because it cannot differentiate an organic from a functional disorder relieved by diazepam.

#### Key Clinical Observations and Disease Progression Based on Sequential, Long-Term Follow-Up Data

Clinical observations in a prospective longitudinal study of 57 anti-GAD-positive SPS patients, probably the largest series of patients examined and followed by the same clinicians every 6 months for a two-year period, have not only confirmed over time the above symptomatology but have also shown that SPS is a progressive disease with worsening clinical picture if untreated [29]. The most common initial symptom in this large series, observed in 68% of the patients when first seen, was the insidious onset of proximal leg stiffness followed by rigidity concurrently in the abdominal, lumbosacral and thoracic paraspinal muscles, lumbar hyperlordosis and impaired gait; 28% of the patients also had various degrees of facial muscle stiffness [29]. About 15% also had ataxia, dysarthria and dysphagia, overlapping with the cerebellar variant, as described below [40]. Diabetes Mellitus Type 1 and other autoimmune diseases, such as vitiligo, pernicious anemia, celiac disease, or thyroiditis, were seen in up to 35% of these patients, confirming data from earlier series of ours and others [3, 8, 17, 18, 33–36]. A ‘startle response’ and an exaggerated reaction to a number of external stimuli were observed in 55 of 57 patients; severe chronic anxiety reactions due to unprotected falls or when expecting physically challenging conditions were seen in 52 of 57 patients. A variety of phobias, such as when walking in crowded places like airports or malls, fear of crossing a street with anxiety of making the duration of the green light or taking escalators were seen in more than 10% of patients; task-specific phobias were also common. Because of the frequent phobias and anxieties, neuropsychiatric testing was performed in collaboration with Mental Health Institute of NIH (NIMH) in ten consecutive patients [41]. It was concluded that the patients perceived their fears as realistic related to the possibility of falls not meeting the DSM-IV criteria for a phobic disorder [41]. Such excessive phobic phenomena are not, however, present in other neurological disorders with spasticity, weakness and falls, like patients with multiple sclerosis and myelopathies, and we still believe that their prominence in patients with SPS-SD is telling us something about their heightened excitability.

Misdiagnoses were also common. Several patients in this large personal series had been earlier diagnosed with conversion or functional disorder because their falls were attributed to avoidant behavior or heightened mental anticipation; others were carrying the diagnosis of myelopathy, dystonia or Parkinsonism, and still others with painful spasms have been on narcotics. It should be pointed out, however, that over-diagnosis of a primary psychiatric or a functional disorder as GAD-negative SPS, especially in patients with non-specific low-GAD titers, remains also an issue as discussed later.

#### Significance of Anti-GAD-Ab Titers, Antigen Recognition and Intrathecal Antibody Synthesis

**Fig. 2:**
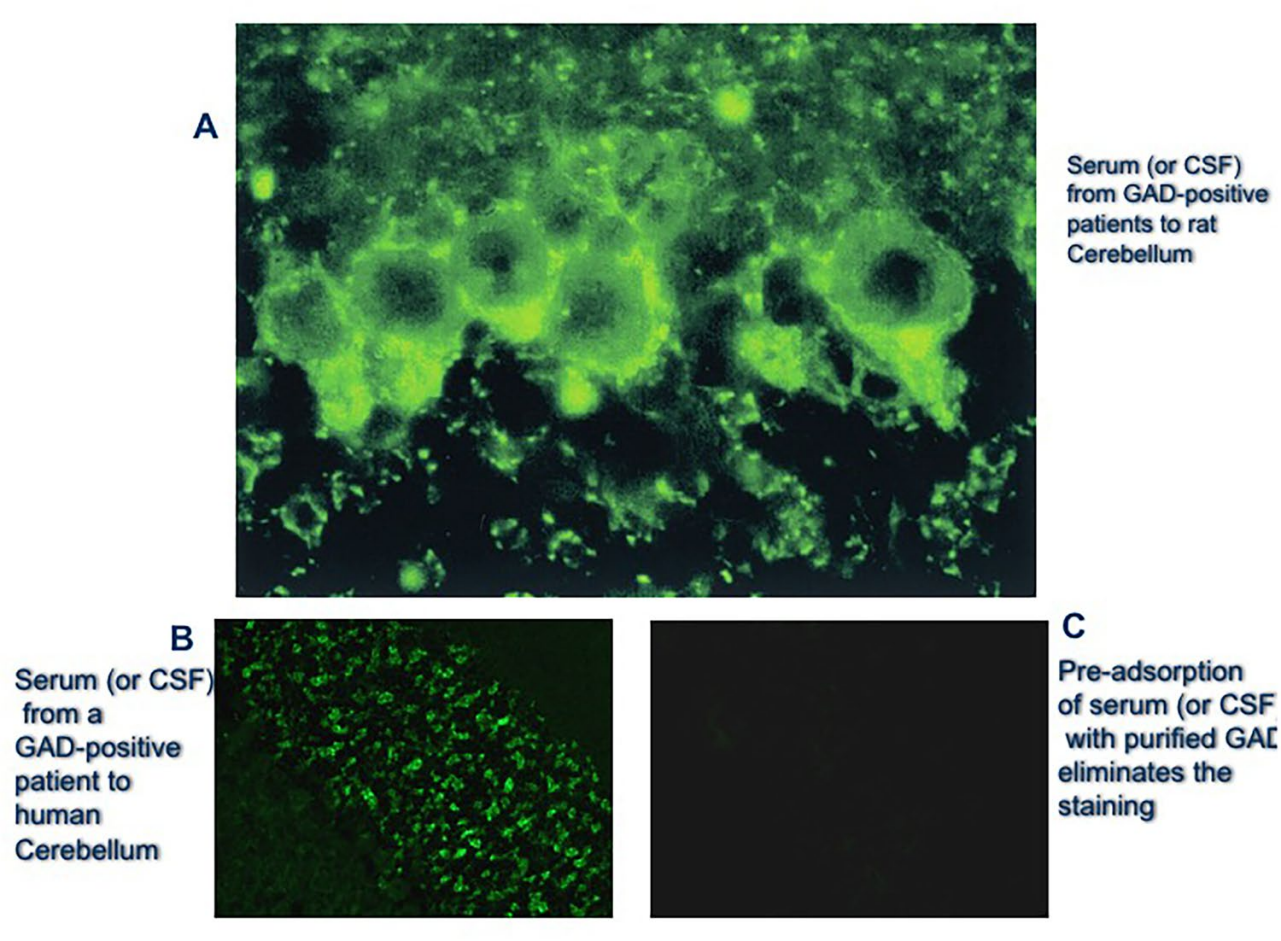
Immunostaining of brain GABAergic neurons with serum or CSF from SPS patients **A**: Immunofluorescent staining of rat cerebellum with serum or CSF from patients with SPS (1:1000 dilution for the serum, 1:25 dilution for CSF; 2-day incubation). Heavy labeling is observed around granule cells in the granular cell layer, in the Purkinje cells, and the molecular layer; the immunostaining pattern precisely corresponds to the distribution of GABA-ergic system in rat brain. **B**, **C**: Similar immunostaining in a larger section of human cerebellum (B) with elimination of staining (C) after pre-adsorption of the patients’ serum or CSF with purified GAD (from Dalakas et al. [18])

**Fig. 3:**
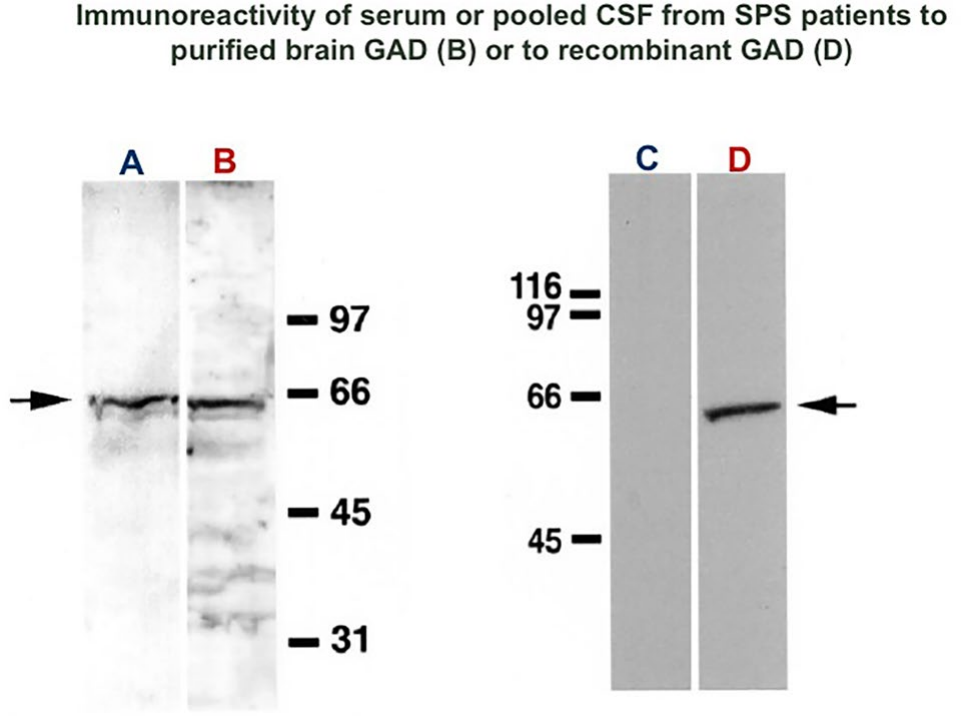
Western blot analysis of GAD65 antibodies from the serum and CSF of patients with SPS and controls. **A**, **B**: Strong immunoreactivity to a 65-kd protein of rat brain extracts is seen with the serum from an SPS patient (B). Mouse monoclonal antibodies against GAD65 recognize the same autoantigen (arrow, A). The molecular weight of protein standards is shown on the right. The same, though weaker, immunoreactivity was obtained with CSF. **C**, **D**: Strong immunoreactivity to purified recombinant GAD65 (arrow D) is seen with the serum from a patient with SPS or with pooled CSF from six SPS patients. No reactivity was seen with pooled serum from patients with insulin-dependent diabetes mellitus (C) (from Dalakas et al. [18])

Since about 20 years ago, serum and CSF anti-GAD65 antibody titers and intrathecal production were measured in 24 SPS patients and 70 disease controls (including 11 with insulin-dependent diabetes mellitus) [18]. All SPS patients with high (> 10,000) anti-GAD65 serum titers also had high CSF titers, from 92 to 2500 ng/mL, and specifically immunoreacted with GABA-ergic neurons on rat cerebellum (Fig. 2A–C) and recognized recombinant GAD65 (Fig. 3) [18]; in contrast, the controlled patients with insulin-dependent diabetes had low serum anti-GAD65 antibody titers and no reactivity to recombinant GAD65 (Fig. 3). CSF oligoclonal IgG bands were detected in 67% of 20 patients tested, with an increased anti-GAD65-specific IgG index present in 85% of patients. Importantly, the mean GABA level in the CSF was lower in SPS patients compared to controls. It was concluded that in SPS: a) there is marked intrathecal antibody response against neuronal GAD65 epitopes [18], indicating clonal B cell activation in the CNS; b) GABA level is reduced in the CSF indicative of impaired GABA synthesis; and c) only high anti-GAD65 antibody titers, confirmed with immunoblots, are highly specific for SPS. This early but fundamental finding on the value of antibody titers has been recently confirmed with concurrent validation by immunohistochemistry and cell-based assay in all GAD-SD patients, highlighting that anti-GAD antibody titers do matter [37]. If the titers by ELISA are high (> 10,000 IU/ml), are diagnostic for a true GAD-SD conferring specificity for an autoimmune neurological disease within the GAD-SD; lower titers (< 10,000 IU/ml) are connected with an atypical or nonspecific neurological disease that may require further investigation, whereas very low titers (< 2,000 IU) are typically seen in diabetes or are of unclear significance.

When GAD antibody titers in the serum are above 10,000, GAD antibodies are also detected in the CSF [18], not requiring a lumbar puncture to ensure specificity, especially in SPS patients where the stiffness in the lumbosacral paraspinal muscles is so severe that requires a radiology-guided procedure. On the other hand, in patients with serum antibody titers below 10,000 or in seronegative GAD-SD, especially patients with encephalitis and those with a seemingly functional disorder resembling SPS, it is essential to test the CSF for GAD antibodies. Of important relevance, GAD antibodies are also detected within the various IVIg preparations as part of the natural antibody repertoire, which means that anti-GAD antibodies are detected in patients receiving IVIg for at least a month after the infusion [42]. Although high titers matter in diagnosis, there is no association between GAD-Ab titer and disease severity and no significantly meaningful titer reduction has been documented after immunotherapies with either IVIg or rituximab based on the two controlled studies [27, 28]. If RIA is used, high titers in SPS-SD are defined as > 20 nmol/L (93% positive) while patients with diabetes without a polyendocrine or autoimmune neurologic syndrome, have titers from 0.03–2.00 nmol/L with cutoff (highest negative value) 0,02 [7, 9, 37, 43].

#### Other Antibodies Connected with SPS and GAD-SD

**Fig. 4:**
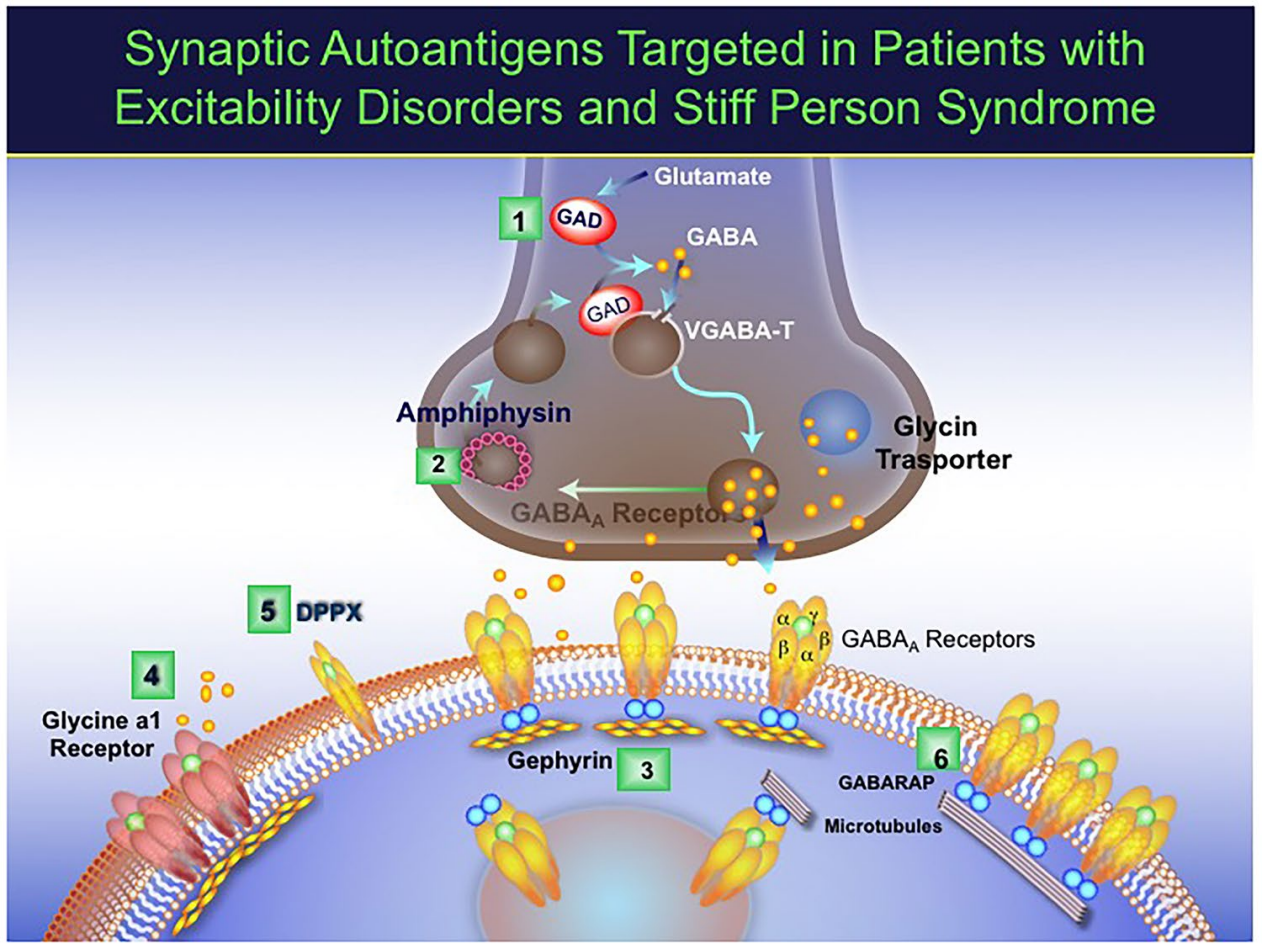
Antigenic targets associated with the inhibitory synapse in patients with SPS-SD. The pre-synaptic antigens are ***GAD*** (1), the enzyme that synthesizes GABA, the main inhibitory neurotransmitter, and ***amphiphysin*** (2), a synaptic vesicle protein responsible for endocytosis of plasma membranes following GABA release. Post-synaptically, the clinically relevant targets within the GAD-SD are: ***gephyri***n (3), a tubulin-binding protein needed for clustering both GABA-A and glycine receptors; ***Glycine Receptor (4)***, a ligand-binding ion channel which allows the passage of chloride ions; and ***DPPX (Dipeptidyl Peptidase-like protein) ***(5), an extracellular regulatory subunit of the Kv4.2 potassium channels on neuronal surface (Kv4.2 complexes have a widespread distribution, not limited postsynaptically as depicted in the figure). Another well identified antigen within the GAD-SD is the ***GABA-A receptor associated protein (GABARAP)*** (6), a linker protein which promotes the organization of the GABAA receptors; anti-GABAR antibodies can be seen in up to 70% of SPS patients [24]. [Modified from Dalakas [34]]

Apart from anti-GAD, other antibodies may be detected in patients with SPS, as depicted in Fig. 4. Autoantibodies against GABA-Receptor-Associated Protein (GABARAP) have been found in about 70% of our patients [24], but on repeated experiments their presence has been inconsistent even if the patients’ sera in vitro inhibits the neuronal expression of GABARAP [24]. Another autoantibody detected in about 10–12% of SPS patients is against glycine-a1 receptor (anti-GlyR), reported the same year by McKeon et al. [44] and our group [45]. In contrast to anti-GAD antibodies, however, the anti-GlyR have a pathogenic role as they recognize extracellular epitopes of the receptor expressed in the spinal cord, brainstem and cerebellum and glycine is a key inhibitory neurotransmitter. Anti-GlyR were first described in PERM (Progressive Encephalomyelitis with Rigidity and Myoclonus) [46, 47], as discussed below. In about 5% of cases, SPS is a paraneoplastic manifestation most often associated with antibodies against amphiphysin [10, 11] and in a single case against gephyrin [12]; in two of our patients with GAD-SPS, the disease was paraneoplastic which means that paraneoplastic SPS can be rarely seen within GAD-SD. Apart from glycine-a1 receptor, all targeted antigens are predominantly cytoplasmic and of unclear pathogenicity; whether they can transiently exhibit an extracellular domain during neurotransmission and exocytosis to exert a pathogenic effect as suggested, remains to be determined [33–35].

### Hyperexcitability of the Motor Cortex, Brain Imaging and Reduction in Brain GABA

To substantiate that impaired GABAergic inhibitory transmission results in cortical hyperexcitability leading to muscle rigidity and spasms, we measured brain GABA and assessed electrophysiologically intracortical inhibition and excitation. Brain MRI imaging is normal in SPS and SPS-SD, but magnetic resonance spectroscopy studies have shown prominent and significant reduction in GABA predominantly in the sensorimotor cortex and to a lesser degree in the posterior occipital cortex, indicating involvement of the inhibitory GABAergic pathways [22]. The reduced brain GABA is also supported by concomitantly finding reduced GABA levels in the cerebrospinal fluid, as mentioned earlier [18].

To determine if GABA reduction in the sensorimotor cortex is associated with dysfunction of supraspinal GABAergic neurons, we performed transcranial magnetic stimulation studies to assess intracortical inhibition and excitation [20]. SPS patients had decreased inhibition and markedly increased facilitation at short intervals pointing to motor cortex hyperexcitability owing to impaired supraspinal GABA-ergic neurons that lead to imbalance between inhibitory and excitatory intracortical circuitry. Such diffuse motor cortex hyperexcitability can produce an excessive corticospinal response upon activation explaining the stimuli-induced muscle spasms seen in SPS. These findings were further supported by the blink reflex studies which showed that the recovery cycle of the R2 component was enhanced in SPS patients compared to controls, indicative of hyperexcitability of brainstem interneuronal circuits due to loss of inhibitory interneurons reflecting a widespread dysfunction of central inhibitory pathways [21].

#### The concept of Reciprocal Inhibition

**Fig. 5:**
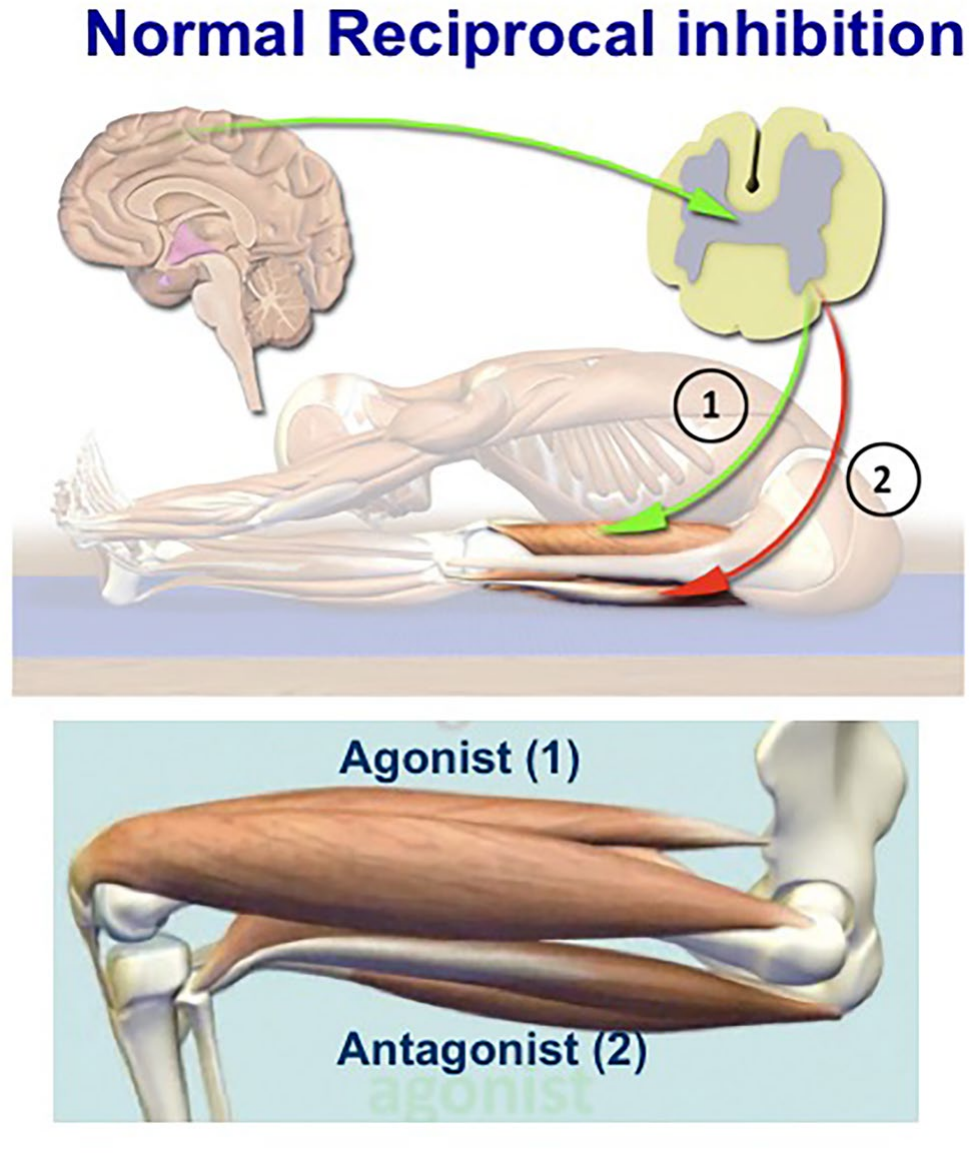
Reciprocal inhibition A: When one muscle is contracted [Agonist (1)], its Antagonist [(2)] is automatically inhibited This is because when CNS sends a message to the alpha motor neurons of the agonists to contract, the inhibitory gamma interneurons of the antagonist muscle interact preventing the opposing alpha motor neurons from firing

**Fig. 6:**
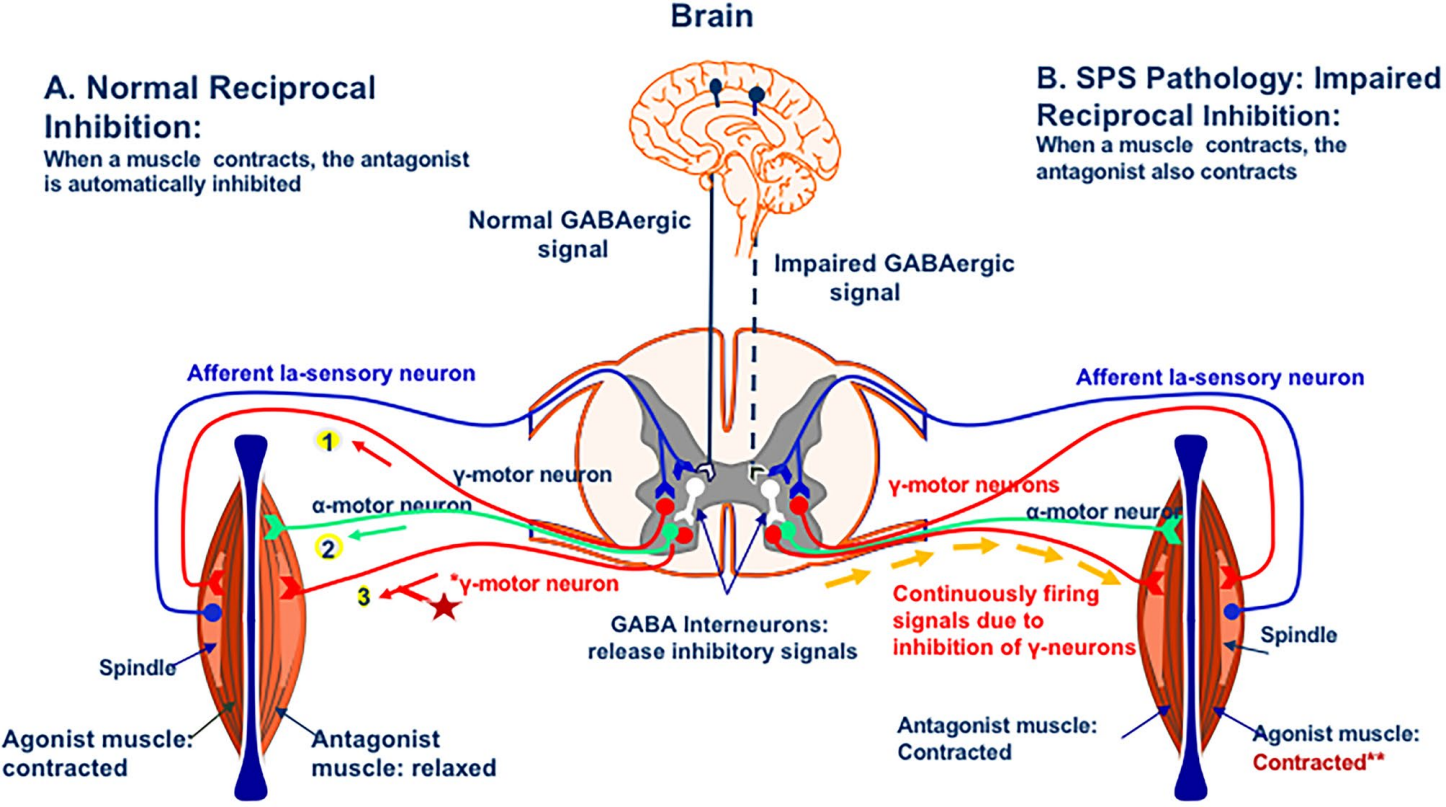
Details of Reciprocal inhibition and stiffness generation in SPS patients: **A**. Normal Reciprocal Inhibition: When an afferent Ia sensory neuron fires, brings information to the spinal cord and stimulates the gamma neuron which, in turn, sends information to the spindle of the agonist muscle to contract (1); when this happens, the gamma motor neuron of the antagonist muscle does not discharge due to inhibition of GABA interneuron (3 **-asterisk**) which, by releasing inhibitory mediators, causes relaxation of the a-motor neuron of the antagonist muscle (2). **C**. Impaired Reciprocal Inhibition results in hyperexcitability and SPS-SD: If the inhibition by the inhibitory GABAergic interneuron is impaired as in SPS, the a-motor neuron is continuously firing and the muscle will be continuously stimulated becoming hypertonic (spastic), without the ability to relax, due to simultaneous contraction of both, the agonist and the antagonist muscles, as highlighted by two asterisks ** [modified from [9]]

Normal physiology is dictated by reciprocal inhibition; this means that when one muscle (such as, biceps) contracts, its antagonist (the triceps) is automatically inhibited (Fig. 5); otherwise, we would have been all stiff. As depicted in Fig. 6, stimulated gamma neurons of an agonist muscle send information to the spindles to contract, while the antagonist’s gamma neurons do not discharge due to inhibition by the inhibitory GABA interneurons, as hypothesized more than 20 years ago [18–21]. When GABAergic neurotransmission is, however, impaired, as occurs in pathologic conditions due to reduced GABA from the cerebral motor pathways, the gamma motor neurons fire continuously because their inhibitory signals are inhibited, resulting in overstimulation of the muscle spindles expressed as simultaneous hypercontraction of agonist and antagonist muscles (Fig. 6); this phenomenon is electrophysiologically detected as continuous motor unit firing in agonist and antagonist muscles and clinically manifested with muscle stiffness [3, 9, 13, 19–21].

#### Immunopathogenic Role of GAD Antibodies

Although GAD antibodies define a clinically heterogeneous group of overlapping GAD-SD [8, 9, 36], all have in common neuronal excitability. It remains, however, uncertain whether GAD antibodies are pathogenic considering they target an intracellular antigen, or they are simply markers of aberrantly activated innate and acquired immunity [33–35]. The observations that anti-GAD65 antibodies in serum and CSF recognize purified GAD antigen by Western blot and specifically immunoreact with GABA-ergic neurons (Fig. 2 and 3), in conjunction with intrathecal synthesis and reduced CSF GABA levels, suggests that the antibodies may be of some functional significance. In vitro, GAD-ab interfere with GABA production [14, 15], while in vivo they affect the function of GABAergic neurons and interfere with GABA synthesis resulting in impaired inhibitory neurotransmission without causing structural brain changes [48–51]. These experimental data are consistent with the MRI imaging, MRS spectroscopy and electrophysiology described above in SPS patients, supporting a neuronal functioning blockade rather than neuronal destruction and explaining the reversibility of the clinical findings seen after therapy, as described later.

GAD exists in two isoforms, GAD65 and GAD67, each encoded by a different gene, with three functional domains, an amino(N)-terminal, a middle PLP-binding, and a carboxy (C)-terminal domain [52]. Patients with SPS-SD show strong immunoreactivity to distinct epitopes compared to DM1 [53–56]; DM1 harbor antibodies against conformational epitopes exclusively located in the PLP- and C-terminals domains, whereas SPS patients predominantly recognize linear epitopes in all three domains having distinct biological effects, compared to DM1 [54–56]. Whether different epitope patterns exist among GAD-related syndromes is, however, still unclear. In our study of 27 patients with diverse GAD-related syndromes, using the previously described high-definition profiling methodology [57], no differences in epitope specificities were found [25]. Others, however, have noted that GAD-Abs from patients with Limbic Encephalitis were more likely to recognize epitopes in the N-terminal domain in contrast to patients with SPS, cerebellar ataxia or epilepsy, that showed more reactivity to the C-terminal domain [48, 58, 59]. Overall, the current data cannot explain the diverse clinical presentation based on different epitope binding patterns.

Additional experimental data have been also informative but not definitive. GAD is found in synaptic vesicles in the nerve endings and is mostly utilized whenever there is an urgent need of GABA synthesis and release [50, 51]. Hippocampal neurons treated with sera from GAD-positive epileptic patients show increased post-synaptic inhibitory potentials [58, 59], while rat cerebellar slices exposed to serum or CSF from patients with SPS or cerebellar ataxia, exhibit decreased post-synaptic inhibitory currents of Purkinje cells [59]. Intracerebral injections of SPS-IgG into rats have also shown a stiffness-like behavior, compared to controls; interestingly, passive transfer studies of GAD-Abs from SPS patients into rats have shown continuous motor activity with increased excitability of anterior horn cells [59, 60]. There is no clear demonstration, however, that anti-GAD antibodies or patients’ sera impair in vivo inhibitory neurotransmission reproducing the main SPS symptoms. This is in contrast to animals treated intraperitoneally or intrathecally with IgG-anti-amphiphysin Abs who have exhibited a clear stiffness-like behavior [61, 62]. Overall, it remains unclear how GAD-Abs can cause GABAergic dysfunction in SPS if they are not internalized into neurons; the possibility, that antigens during synaptic transmission transiently expose extracellular epitopes which are then recognized by the immune system, remains still hypothetical [9].

In patients with GAD-antibody-related neurological syndromes [63], circulating GAD-reactive B cells that can differentiate into antibody producing cells have been detected in the peripheral blood and bone marrow, suggesting that targeting memory B cells (i.e., with rituximab) or plasma cells (i.e., with. bortezomib), may have therapeutic implications in SPS-SD [63].

### GAD-Positive Cerebellar Ataxia

Anti-GAD antibody-associated cerebellar ataxia is the second most frequently seen GAD-related neurological disorder [38]. It affects more women than men, often with comorbid diabetes or polyendocrine autoimmunity [3–9, 40, 64]. Patients exhibit gait and limb ataxia, nystagmus, often severe dysarthria, and oculomotor dysfunction, most often overlapping with the typical SPS symptomatology that worsens the overall clinical picture. CSF can show oligoclonal bands and intrathecal anti-GAD antibody synthesis [18, 40]. Importantly, there is no cerebellar atrophy on the MRI imaging, except of mild changes in rare instances [40], implying a functional blockade of cerebellar pathways rather than a destructive neuronal process [18, 40].

As discussed earlier, it is unclear whether the antibodies play a role in the pathogenesis of cerebellar ataxias. A monoclonal GAD65 antibody has been shown to interfere with GABAergic neurotransmission in brain slice preparations and elicits in animals neurophysiological and behavioral effects mimicking cerebellar ataxias [49, 64]. Intracerebellar administration of IgGs from CSF of patients with GAD-associated cerebellar ataxia can impair cerebellar modulation of motor control but whether it contributes to patients’ poor coordination is unclear [49, 64–66]. The anti-GAD antibodies may also act on nerve terminals of GABAergic interneurons depressing the release of GABA resulting in neuronal hyperexcitability but, whether this process can eventually disturb the function of Purkinje cells, as proposed [65, 66], remains hypothetical. Considering that cerebellar ataxia is disabling, there is a need to explore if GAD-antibody pathogenicity is the main responsible process to design specific pharmacological or even neurostimulating therapies.

### GAD-Positive Autoimmune Epilepsy

Anti-GAD antibodies are seen in patients with pharmaco-resistant epilepsy, most often temporal lobe epilepsy [67–69]. Some patients present with refractory convulsive and non-convulsive status epilepticus with frequent autoimmune comorbidities but normal MRI [70–74], In a retrospective series, anti-GAD antibodies were detected in 22% of patients with various epilepsies and concurrent autoimmune comorbidities [72]. Among 233 patients with all types of epilepsy, 2,3% had GAD-abs but, if only patients with focal epilepsy are considered, GAD-Abs were present in 16% of all cases [75]. In other series, among patients with temporal lobe epilepsy the percentage of GAD-antibody positivity may be even higher up to 21,7% [74]. Of interest, among 80 children with epilepsy, anti-GAD antibodies were the third most common antibody, after antinuclear and anti-Voltage Gated Potassium Channels [74]. At least 5% of SPS patients have seizures but, in our experience, the epilepsy in SPS is not refractory but rather easily controlled. Musicogenic reflex seizures, although rare, have been more frequently noted among patients with GAD-associated epilepsy [74]. In a recent retrospective chart review of 16 patients with musicogenic epilepsy, 9 tested patients were found to be GAD-antibody positive in both serum and CSF [76]. These patients had temporal lobe epilepsy with epileptiform EEG abnormalities captured when seizures were induced by music [76]; only one of 6 patients partially responded to immunotherapy raising doubts as to whether this epilepsy is of immune etiology.

The mechanism of GAD-epilepsy is unclear, but a reasonable hypothesis is the association of anti-GAD antibodies in inducing hyperexcitability by inhibiting GABAergic pathways. The intrathecal synthesis of GAD antibodies may also affect GABAergic pathways and decrease the conversion of glutamate to GABA resulting in excessive excitatory neurotransmission that lowers the seizure threshold [9]. Because cytotoxic T lymphocytes have been found in temporomesial tissue biopsies from some GAD-positive patients with pharmaco-resistant epilepsy, a cellular neurotoxic effect against GABAergic interneurons has been also implicated [9, 32].

### GAD-Positive Limbic Encephalitis

Autoimmune limbic encephalitis with anti-GAD antibodies clinically presents like the classic autoimmune or paraneoplastic limbic encephalitides with impaired working memory, psychiatric symptoms, seizures or altered level of consciousness [6–9, 30–32]. Like the other GAD-SD, the causative role of GAD antibodies is still unclear. Some patients have oligoclonal CSF bands and intrathecal GAD-antibody synthesis.

### Progressive Encephalomyelitis with Rigidity and Myoclonus (PERM)

PERM, described the same year as SPS, is also considered as an SPS-spectrum disorder [77]. PERM is, however, a distinct syndrome characterized by muscle spasms and stiffness, gait ataxia, myoclonic jerks, a varying degree of brainstem dysfunction with oculomotor abnormalities and dysphagia, prominent autonomic symptoms and depressed level of consciousness. PERM is equally present in men and women although in our small series most patients were men. The disorder is characterized by the presence of anti-GlyR antibodies, which are also detected in up to 15% of GAD-positive SPS patients [44, 45, 78, 79]. An underlying tumor, especially thymoma or lymphoma, can be present in about 20% of PERM patients [78]. Rare histological data have demonstrated inflammatory and microglial changes as well as neuronal cell loss in the pons, medulla, cerebellum, spinal cord and autonomic ganglia [9]. Another autoantibody that has been detected in 4 patients with PERM is against dipeptidyl-peptidase**-**like protein (DPPX), a regulatory subunit of neuronal Kv4.2 potassium channel complex responsible for transient inhibitory currents that regulate repetitive firing rates into neuronal dendrites [80, 81] (Fig. 4). Because of the widespread distribution of Kv4.2 complexes, these patients present with a multifocal neurologic phenotype including prominent gastrointestinal manifestations with weight loss and diarrhea, cognitive dysfunction, memory deficits, CNS hyperexcitability, myoclonus, tremor, seizures, encephalopathy, sleep disturbance and dysautonomia.

### GAD-positive Nystagmus and Abnormal Eye Movements

Isolated oculomotor dysfunction can be the sole manifestation in some patients with anti-GAD antibodies, highlighted by downbeat or horizontal nystagmus and saccadic intrusions or oscillations. In our experience, oculomotor dysfunction is not unusual among all GAD-positive SPS patients especially those with cerebellar ataxia [29, 32, 33]. The most common isolated GAD-positive oculomotor dysfunction is persistent horizontal or downbeat nystagmus, presumably related to excitability of vestibular nuclei with increased drive to motor neurons of ocular musculature, resulting in an upward slow phase followed by a quick compensatory downward phase [83–87]. Within the spectrum of GAD-antibody-associated abnormal eye movements, opsoclonus and myoclonus have been also observed [29, 82–88].

## Therapeutic interventions in SPS

For SPS, two treatment strategies are implemented, *symptomatic* or *immunologic,* either independently or in combination, depending on symptom severity [9, 33, 34, 82, 89, 90].

**Fig. 7:**
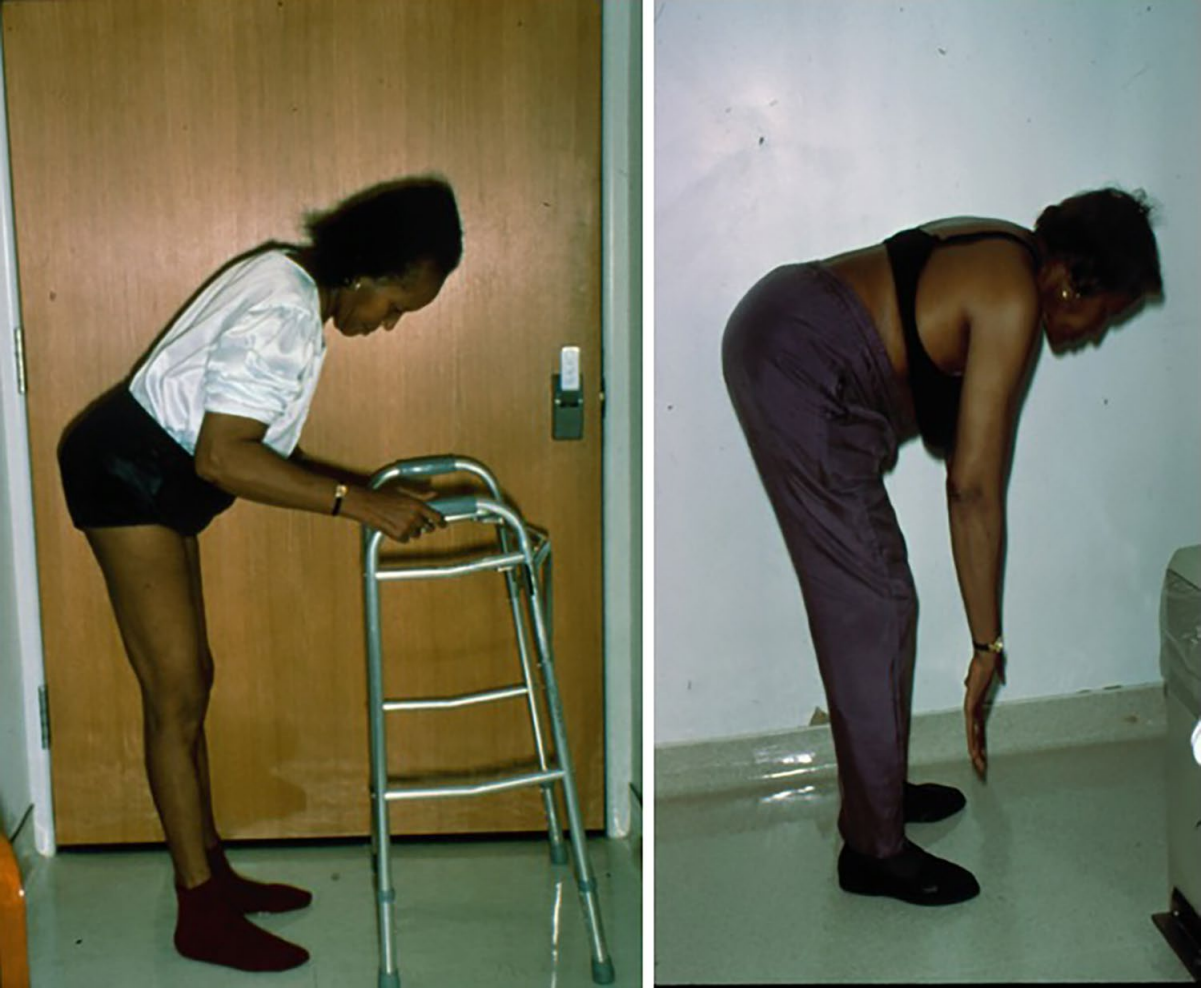
Improvement of the stiffness and walking ability of an SPS patient 3 months after IVIg therapy. Before treatment (left) and after IVIg (right) [based on the IVIg controlled trial (Dalakas et al. [27])**]**

A ***Symptomatic Therapy.*** This is based on diverse agents that all enhance GABAergic neurotransmission and remain the hallmark therapy for the disease and the first-line drugs for therapy initiation, although no controlled studies have been ever conducted. They include:
  i) *Benzodiazepines*. They are all GABAA agonists, with diazepam being the oldest and most effective therapeutic option since the description of SPS [1]. These family of drugs can help most patients, although the high doses sometimes required cannot be tolerated and may lead to addiction. We start with diazepam 5–10 mg BID and, if well tolerated, increase it to TID. Similar compounds include clonazepam, alprazolam, lorazepam and temazepam. Diazepam is especially effective in status spasticus and, if needed, it is more effective intravenously;
  ii) *Anti-spasticity agents.* They are mostly GABAB agonists, with *baclofen* being the most effective. Between benzodiazepines and anti-spasticity agents, baclofen is our first treatment option because, compared to diazepam, is better tolerated and does not lead to addiction. We start with 10 mg TID but sometimes higher doses may be used for better efficacy, even up to 50 mg daily, but caution is needed because of cognitive side effects. Some SPS patients have been treated with baclofen pump intrathecally to improve spasticity; we have not initiated such therapy in our patients because we have been overall disappointed with the results witnessed in several patients already on baclofen pump, referred to us. Our overall impression is that the benefit of the pump is marginal and the complications more significant, especially if the patients also have DM1 and use insulin pump or receive subcutaneous IgG.
  iii) *Antiepileptics*. These can also enhance GABAergic neurotransmission and improve SPS symptomatology, in conjunction with baclofen and benzodiazepines. In our experience, the most helpful agents in this family are gabapentin and vigabatrin, which act by inhibiting GABA-transaminase [82, 89]. Tiagabine, an inhibitor of GABA reuptake, and levetiracetam, which facilitates inhibition of GABAergic transmission may offer benefits, if well tolerated. Other drugs include tizanidine, a centrally acting α2 adrenergic receptor, and dantrolene, a muscle relaxant, that sometimes may be useful.
  iv) *Botulinum Toxin.* It can provide short-term benefits and may be considered for some patients with localized spasms or prominent painful spasms in one extremity (stiff-leg syndrome) or the lumbosacral spine, if have been unresponsive to the other therapies. The benefit, based on our experience, has been overall minimal and short-lived, while the doses required are quite significant for routine use.
  v) *Supportive therapies.* SPS patients experience severe anxiety due to phobias of falling or completing even simple physical tasks and many times require psychological support both at home or at work especially if the symptoms are significant and do not concurrently improve with the physical symptomatology. Their phobias often lead to depression, while their painful spasms may at times lead to addiction of drugs like benzodiazepines or narcotics, highlighting the need for multifactorial care from the outset.
B ***Immunotherapy***
  If the above agents do not offer a satisfactory benefit, we proceed to immunotherapy, which is sequentially as follows:
  - *Intravenous immunoglobulin (IVIg)*. This is the first in line agent in this category based on its proven efficacy and excellent tolerance. In a randomized, double-blind, placebo-controlled trial, we conducted in GAD-positive SPS patients, IVIg resulted in significant improvements in objective stiffness parameters, hyperexcitability and activities of daily living [27], based on validated quantitative scales. The drug clearly improves stiffness and muscle flexibility especially in the paraspinal muscles (Fig. 7), improves gait preventing falls and reduces even anxiety-triggered spasms. In the patients that first received IVIg, the stiffness scores significantly decreased (p = 0.02) and the heightened-sensitivity scores markedly declined, but all rebounded when the patients were switched to placebo; the opposite occurred in patients randomized first to placebo and switched then to IVIg [27]. Overall, the patients who received IVIg compared to placebo were able to walk without assistance or falls and perform daily activity functions. This pivotal study has clearly shown that IVIg for up to 3 months is effective in SPS patients not adequately responding to anti-spasmodic and GABA-enhancing drugs [27, 91]. The dose of IVIg is based on 2 g/kg, divided in 2–5 consecutive days according to the patient’s age, co-morbidities or total weight. In many controlled studies, the total IVIg dose was divided in 2 days; in practice, it is often divided in 3–5 days especially when given as home-infusion to ensure safety and better tolerance. In overweight patients, the ideal-body weight is used to calculate the total dose, as described [92]. The duration of efficacy after each monthly IVIg infusion ranges from 4 to 5 weeks, and repeated infusions may be required in several patients with a preferred maintenance dose of 1 g/kg. The long-term monthly maintenance therapy of IVIg for chronic SPS management has not been, however, tested in a controlled study resulting sometimes in overuse as dependency test is not routinely used and many patients require it for long-time periods out of fear that may worsen without it.
    To ensure the judicious use of IVIg, we advise the patients from the outset that after the first 3 monthly infusions, if there is an objective benefit, we will continue with periodically performing dependency tests by reducing the IVIg dose or prolonging the infusion intervals to objectively assess regression in severity of spasms and stiffness, as recently highlighted [92]. Unfortunately, sometimes IVIg is being used just to reduce pain and improve fatigue or other subjective symptoms; a conditioning effect is also prevalent, as recently highlighted [92], with patients requesting therapy continuation out of fear they may worsen if it is stopped. IVIg still, however, remains the only immunomodulatory therapy with proven benefit in SPS patients, but there is a need to determine long-term benefits beyond the originally tested 3-month period [26]. We are currently in the process of assessing long-term benefits over a 10-year period. Subcutaneous immunoglobulin may be also an option in patients with poor venous access or when there is a demonstrable early wearing-off effect to ensure sustained benefit [92, 93].
  - *Rituximab.* If IVIg is not sufficiently effective or totally ineffective, we proceed to *Rituximab* which anecdotally has shown some benefit [94]. A randomized controlled trial we conducted in SPS patients demonstrated lack of efficacy of rituximab compared to placebo owing to a strong placebo effect [28]. In this series, however, 7 patients improved and four of them with severe disease demonstrated meaningful to impressive improvements. On this basis, rituximab is a useful drug for a subset of patients who have failed therapies with GABA-enhancing drugs and IVIg. Anti-GAD antibody titers may drop, but not at a statistically significant level [27, 28]; as mentioned earlier, antibody titers do not correlate with clinical severity or predict improvement.
    The need for follow-up Rituximab infusions for those who have initially responded remains empirical. In patients who improved and have been stable, we wait for new worsening signs which sometimes can be seen as late as 1–3 years later; for those, however, who regress earlier, after 6–8 months, we use 2 g every 6–12 months or 1-g every 3–6 months to ensure stability [95, 96]. The CD27 + memory B-cells may be a useful biomarker to follow because this B cell subpopulation correlates best with stability when their counts are at or below the therapeutic target, or with disease worsening upon their reemergence [95, 96]. We do not follow routinely the CD19/20 B cells, but we do follow the IgG immunoglobulin levels every 3–6 months because, if below normal, patients may be susceptible to infections or prolonged recovery after infectious illnesses especially during the COVID19 pandemic. Rituximab has been also effective in one of our patients with PERM associated with Glycin-Receptor antibodies [97]. This patient, who was hospitalized in ICU for 12 months, requiring mechanical ventilation and being unresponsive to IVIg or other therapies, started to improve after the first Rituximab infusion with concurrent reduction in the Glycin-receptor antibodies in serum and CSF; he became able to walk after the second infusion while antibodies became undetectable in the CSF although still detectable at low titers in serum [97].
  - *Autologous hematopoietic stem cell transplantation (auto-HSCT).* Auto-HSCT has been used in patients with severe SPS who failed conventional immunosuppressive therapy, with variable results*.* In a small study, 3 patients with SPS and one with PERM, initially treated with Cyclophosphamide (Cy) 2 g/m2 + G-CSF and then conditioned with Cy 200 mg/kg + ATG followed by auto-HSCT, exhibited improved ability to perform for physical tasks [98]. The walking distance of one patient improved from 300 to 5 miles while one other’s ambulation improved from being wheelchair-bound to being able to walk with a walker; two patients became seronegative for anti-GAD antibodies and their neurophysiological abnormalities were normalized [98]. Although auto-HSCT was suggested as a treatment option for some SPS patients refractory to conventional immunotherapy, a large study aiming at 40 SPS patients was terminated early after enrolling 23 patients because of no efficacy or only transient benefits, taking into account potential serious complications [99]. One of the several limitations of that study was the recruitment of patients with advanced disease; considering the strong placebo effect as noted in the rituximab trial, the need for using objective measurements in quantifying stiffness with validated scales were pointed out [100]. Whether, a controlled HSMT trial will be meritorious in SPS patients with early disease unresponsive to therapies remains uncertain, taking into account the potential side effects.
  - *Other partially effective or failed therapies*. *Plasmapheresis* has been tried and some patients may respond based on small anecdotal case reports, [101] but in our experience it has limited and transient benefits and we do not routinely use it. *Corticosteroids* have been surprisingly ineffective based on our experience while triggering or exacerbating diabetes may also be a consideration for possible long-term use. Intravenous steroids, although seemingly effective in GAD-associated encephalitis, are overall disappointing in SPS even in the acute spastic state (status spasticus). Good control of diabetes, especially when requires insulin, remains a critical factor because, if uncontrolled, seems to worsen the neurologic symptomatology. *Immunosuppressive* agents such as azathioprine, methotrexate, cyclophosphamide, and mycophenolate mofetil used for maintenance in other autoimmune neurological diseases have been also disappointing, in spite of rare case reports.

## Therapeutic Interventions in Patients with SPS-Plus Cerebellar Disease

The reason this disease subset requires specific mentioning is because these patients do not adequately respond to all the aforementioned therapies even if the treatment algorithm remains the same. Based on our experience with more than 10 treated patients, we have noticed that some patients partially respond to IVIg or rituximab early in their disease course but in patients with longer-standing disease the cerebellar component, especially the dysarthria and dysphagia, seems to progress and dominates the clinical symptomatology over time. Although the SPS component in some patients may continue to partially respond to IVIg or rituximab, the ataxia, dysarthria and dysphagia continue to slowly progress. Of note, early in the disease the brain MRI of these patients is usually normal, but over time mild signs of cerebellar atrophy may become apparent probably explaining the clinical progression.

## Therapeutic Interventions for GAD-Autoimmune Epilepsy and Limbic Encephalitis

Therapy in autoimmune epilepsy and acute or subacute autoimmune limbic encephalitis, starts with IV steroids 1,000 mg daily for 3–5 days, followed by IVIg and rituximab as needed. Anti-epileptics are added in patients with epilepsy, but many patients may not need them several months after they have fully improved.

## A view to Potential Future Immunotherapies

Novel therapeutic approaches that need to be evaluated in SPS should include monoclonal antibodies against B cells or plasma cells based on the assumption that SPS-SD are antibody-mediated diseases and antibody-producing B cells or plasmablasts are presumably activated. Considering the significant disability some of these patients have and the steady disease progression, the following promising anti-B cell agents might be important considerations, as recently highlighted [102, 103]: 1) other anti-CD19/20- B cells currently on the market, some of which are already approved in neurological autoimmunities, such as *Ocrelizumab, ofatumumab, Inebilizumab* and *Obexelimab (XmAb5871*)*. Inebilizumab* also targets antibody-producing CD-19-positive plasmablasts and plasma cells while *Obexelimab* not only targets CD19 but binds simultaneously to both CD19 and Fc**γ**RIIb promoting internalization of CD19 in the lipid rafts, markedly enhancing the inhibitory FcγRIIB and downregulating CD19 as proposed for the IgG4-neurological autoimmunities [102–104]; 2) *bortezomib* that targets plasmblasts; and 3) *Zanubrutinib and Rilzabrutinib*, both Bruton’s tyrosine kinase inhibitors, showing already promise in patients with multiple sclerosis [103]. FcRn inhibitors, such as the recently approved Efgartigimod [104, 105], may be an additional family of agents that act by enhancing the catabolism of circulating IgG antibodies. Finally, the IL6-Receptor antagonists such as Satralizumab and Tocilizumab approved for NMO-SD [103] that also show promise in NMDAR-encephalitis [106] may be additional therapeutic options that need to be tested in controlled studies.

## Supplementary Information

Below is the link to the electronic supplementary material.

Supplementary file1 (PDF 611 kb)
